# Risk Factors and Pregnancy Outcomes of Twin Pregnancies With Gestational Diabetes Mellitus: A Comparison Based on Chorionicity

**DOI:** 10.1155/jdr/9744892

**Published:** 2026-01-13

**Authors:** Shuai Huang, Xiaoyan Li, Jing Zheng, Gao Qiu, Ping Luo, Lu Mao, Hongbo Qi

**Affiliations:** ^1^ Department of Obstetrics, The First Affiliated Hospital of Chongqing Medical University, Chongqing, China, cqmu.edu.cn

**Keywords:** chorionicity, gestational diabetes mellitus, pregnancy outcomes, risk factors, twin pregnancies

## Abstract

**Background:**

There is insufficient evidence to determine whether the risk factors and pregnancy outcomes associated with gestational diabetes mellitus (GDM) in twin pregnancies vary by chorionicity.

**Materials:**

A retrospective cohort study was conducted among twin pregnancies. GDM was diagnosed using the IADPSG diagnostic criteria. Logistic regression and generalized estimation equation (GEE) models were used to identify the risk factors of GDM and its impact on pregnancy outcomes, stratified by monochorionic (MC) and dichorionic (DC) pregnancies.

**Results:**

Advanced maternal age (MC: aOR 2.18, 95% CI 1.25–3.81 and DC: aOR 1.32, 95% CI 1.06–1.67) and preexisting hypertension (MC: aOR 2.69, 95% CI 1.04–9.36 and DC: aOR 1.70, 95% CI 1.12–2.59) were risk factors for GDM regardless of chorionicity. Overweight (aOR 1.65, 95% CI 1.26–1.98), obesity (aOR 2.31, 95% CI 1.43–3.74), multiparity (aOR 1.43, 95% CI 1.10–1.88), assisted reproductive technology (ART) use (aOR 1.75, 95% CI 1.36–2.26), and polycystic ovary syndrome (PCOS) (aOR 1.98, 95% CI 1.37–4.12) were risk factors for GDM only in DC pregnancies. GDM was only associated with an increased risk of preeclampsia in MC pregnancies (aOR 1.29, 95% CI 1.04–2.26). GDM was associated with an increased risk of preterm delivery (PTD) at < 37 (aOR 1.13, 95% CI 1.05–1.34) and < 34 gestational weeks (aOR 1.15, 95% CI 1.07–1.75) in DC pregnancies.

**Conclusion:**

The risk factors and pregnancy outcomes associated with GDM in twin pregnancies vary by chorionicity.

## 1. Introduction

Gestational diabetes mellitus (GDM) is defined as a glucose intolerance with onset or first recognition during pregnancy [1]. The global prevalence of GDM has steadily increased over the past few decades, primarily due to the rising maternal age, the obesity epidemic, and the changes in diagnostic criteria [2]. GDM is associated with adverse maternal outcomes, including preeclampsia (PE), cesarean delivery, and subsequent type 2 diabetes, as well as neonatal complications such as macrosomia, hypoglycemia, hyperbilirubinemia, and shoulder dystocia [3].

With the development of assisted reproductive technology (ART) and advanced maternal age, twin pregnancies now account for approximately 3%–4% of all births, a significant increase over the past 3 decades [4]. Twin pregnancies are inherently high risk, exhibiting elevated rates of maternal complications including GDM, PE, gestational hypertension (GH), anemia, postpartum hemorrhage (PPH), and cesarean delivery [5–7]. Twin pregnancies are classified based on the chorionicity as either monochorionic (MC), sharing a single placenta, or dichorionic (DC), having two separate placentas [8]. Previous studies have demonstrated that chorionicity has a modifying effect on influencing factors and pregnancy outcomes in twin pregnancies [9–13]. In addition, distinct patterns in maternal serum analytes, placental function, and fetal growth were found between MC and DC pregnancies [14].

Although numerous studies have investigated the associations between GDM and pregnancy outcomes in twin pregnancies [15–28], comparisons of pregnancy outcomes complicated by GDM between MC and DC pregnancies remain limited. Understanding how chorionicity influences the development of GDM and its outcomes is crucial for several reasons. First, the differing placental structures and functions between MC and DC pregnancies may affect glucose metabolism differently [29]. Second, the baseline risks for adverse outcomes vary by chorionicity, potentially altering the additional impact of GDM. Third, management strategies may need to be tailored based on both GDM status and chorionicity to optimize outcomes.

In light of these considerations, this study is aimed at addressing this knowledge gap by investigating the risk factors associated with developing GDM and the pregnancy outcomes related to GDM in twin pregnancies, stratified by chorionicity.

## 2. Methods

### 2.1. Data Source and Study Participants

This retrospective cohort study enrolled twin pregnant women who gave birth at the First Affiliated Hospital of Chongqing Medical University from January 2016 to December 2022. Twin pregnancies were identified by searching the hospital information system (HIS). The exclusion criteria were (1) intrauterine death of one or both fetuses; (2) structural or genetic anomalies of one or both fetuses; (3) severe fetal complications, such as twin‐to‐twin transfusion syndrome, twin reversed arterial perfusion sequence, and twin anemia–polycythemia syndrome; (4) delivery < 28 weeks of gestation; (5) preexisting diabetes mellitus or autoimmune diseases; (6) missing GDM screen; (7) unknown chorionicity; and (8) incomplete baseline information. The study protocol was approved by the Ethics Committee of the First Affiliated Hospital of Chongqing Medical University (No. 201530), and the requirement for informed consent was waived due to the retrospective nature of the study. All works were conducted in accordance with the Declaration of Helsinki (1964) and Strengthening the Reporting of Observational Studies in Epidemiology (STROBE) checklist.

### 2.2. Diagnosis of GDM

All twin pregnant women underwent a standard 75‐g oral glucose tolerance test (OGTT) between 23 and 28 weeks of gestation. GDM was diagnosed according to the International Association of Diabetes and Pregnancy Study Groups (IADPSG) criteria, which defines GDM as the presence of one or more of the following thresholds: fasting plasma glucose ≥ 5.1 mmol/L, 1‐hour plasma glucose ≥ 10.0 mmol/L, or 2‐hour plasma glucose ≥ 8.5 mmol/L [30]. Women who were diagnosed with GDM received standard care including dietary counseling, blood glucose monitoring, or insulin therapy when indicated.

### 2.3. Data Collection and Definitions

We collected maternal demographic and clinical characteristics including age, pre‐pregnancy weight, height, pre‐pregnancy body mass index (BMI), gravidity, parity, mode of conception (spontaneous vs. ARTs use), chorionicity, history of present illness, history of previous pregnancy complications, family history of diabetes mellitus, thyroid function in the first trimester, GDM treatment, and gestational weight gain. Maternal outcomes including GH, PE, intrahepatic cholestasis of pregnancy (ICP), gestational age at delivery, premature rupture of membranes (PROM), mode of delivery, and PPH. Neonatal outcomes including birthweight, large for gestational age (LGA), small for gestational age (SGA), Apgar scores at 5 min, neonatal hypoglycemia, and admission to neonatal intensive care unit (NICU).

Maternal age was categorized into < 35 years and ≥ 35 years. Maternal BMI was calculated as weight in kilograms divided by height in meters squared and categorized into underweight (BMI < 18.5 kg/m^2^), normal weight (18.5–23.9 kg/m^2^), overweight (24.0–27.9 kg/m^2^), and obesity (≥ 28.0 kg/m^2^) based on China′s BMI standard. Chorionicity was determined by first‐trimester ultrasound examination based on the lambda or T‐sign at the inter‐twin membrane insertion [8]. In cases where first‐trimester ultrasound was unavailable, chorionicity was determined based on placental histopathological examination after delivery. Gestational weight gain was calculated as the difference between predelivery weight and pre‐pregnancy weight. Hypothyroidism in the first trimester was defined as thyroid‐stimulating hormone (TSH) concentration ≥ 2.5 mIU/L [31]. Hyperthyroidism in the first trimester was defined as TSH concentration < 0.1 mIU/L [32]. Preexisting hypertension was defined as hypertension before pregnancy or less than 20 weeks of gestation. GH was defined as new onset of hypertension (systolic blood pressure ≥ 140 mmHg and/or diastolic blood pressure ≥ 90 mmHg on at least two occasions, at least 4 h apart) after 20 weeks of gestation in a previously normotensive woman, without proteinuria or other systemic features of PE [33]. PE was defined as GH with proteinuria (≥ 300 mg in 24 h or protein/creatinine ratio ≥ 0.3 mg/mg, or dipstick reading ≥ 2+) or end‐organ dysfunction after 20 weeks of gestation [33]. ICP was defined as elevated serum total bile acid ≥ 10 *μ*mol/L with pruritus [34]. PTB was divided into < 37, < 34, and < 32 gestational weeks. PROM was diagnosed when spontaneous rupture of membranes before the onset of labor, regardless of gestational age [35]. PPH was defined as blood loss ≥ 500 mL within 24 h after vaginal delivery, or ≥ 1000 mL after cesarean delivery [36]. LGA and SGA were defined as a birth weight higher than the 90th percentile or below the 10th percentile for gestational age and sex according to Chinese twin birth weight reference [37]. Neonatal hypoglycemia was defined as blood glucose level of < 2.2 mmol/L within the first 24 h and requiring medical intervention [38].

### 2.4. Statistical Analysis

Continuous variables were presented as mean (standard deviation) or median (interquartile range) based on their distribution, which was assessed using the Shapiro–Wilk test. Categorical variables were presented as numbers and percentages. For comparisons between groups (GDM vs. non‐GDM), Student′s *t*‐test or Mann–Whitney *U* test was used for continuous variables as appropriate, and chi‐square test or Fisher′s exact test was used for categorical variables. Multivariable logistic regression analysis was performed to identify independent risk factors for developing GDM and the impact of GDM on pregnancy outcomes. Potential confounders in the multivariable models included maternal age, maternal BMI, multiparity, ART use, preexisting hypertension, polycystic ovary syndrome (PCOS), history of GDM, hypertensive diseases of pregnancy, insulin treatment, and gestational weight gain. GEE model was used to assess the association between GDM and neonatal outcomes to address the inter‐twin correlation. Odds ratios (ORs) with 95% confidence intervals (CIs) were calculated. All analyses were performed stratified by chorionicity.

A two‐sided *p* value < 0.05 was considered statistically significant. All statistical analyses were performed using the SPSS Version 26.0 (IBM Corp., Armonk, New York, United States).

## 3. Results

### 3.1. Characteristics of the Study Participants

**Figure 1:**
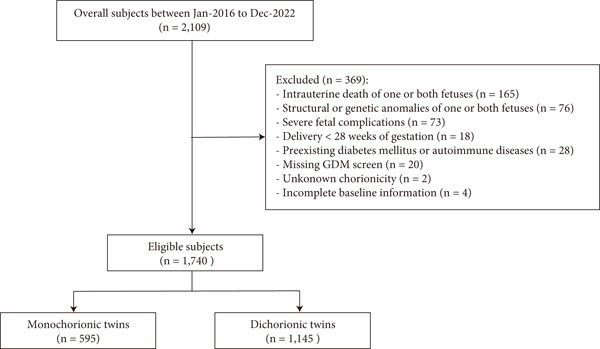
Flowchart of selecting participants in the study.

Figure 1 illustrates the selection of participants. After excluding women who met the exclusion criteria, a total of 1740 participants were included in the final analysis, comprising 595 MC and 1145 DC twin pregnancies. The baseline characteristics of the excluded and included participants were similar, except for a higher incidence of GDM in the final participants, as women without GDM screening were excluded (Table S1). Among the included population, the overall prevalence of GDM was 26.9% (*n* = 160) in MC pregnancies and 30.0% (*n* = 343) in DC pregnancies. The proportion of maternal age ≥ 35 years, overweight, conceived with ART, and complicated with PCOS was higher in DC pregnancies (Table S1). Although the difference in GDM prevalence between chorionicity groups was not statistically significant, postload glucose levels were higher in DC pregnancies (Table S2).

The characteristics of the study participants, stratified by chorionicity and GDM status, are presented in Table 1. In both chorionicity groups, women with GDM were more likely to be maternal age ≥ 35 years and conceived with ART compared with those without GDM. The mean pre‐pregnancy BMI and gestational weight gain were higher in women with GDM in both MC and DC pregnancies. Overweight, obesity, multiparity, preexisting hypertension, and complicated with PCOS were more prevalent in women who developed GDM, especially in DC pregnancies.

**Table 1:** Characteristics of the study participants.

**Variables**	**MC**			**DC**		
	**No GDM (** **n** = 435**)**	**GDM (** **n** = 160**)**	**P** **value**	**No GDM (** **n** = 802**)**	**GDM (** **n** = 343**)**	**P** **value**
Maternal age, y	29.0 (4.0)	30.1 (4.4)	< 0.001	30.9 (3.7)	31.9 (3.5)	< 0.001
≥ 35	39 (9.0)	32 (20.0)	0.001	128 (15.9)	77 (22.4)	0.009
Pre‐pregnancy BMI, kg/m^2^	20.8 (2.6)	22.0 (3.2)	< 0.001	21.4 (2.7)	22.4 (3.0)	< 0.001
Underweight (< 18.5)	85 (19.5)	14 (8.8)	0.001	97 (12.1)	24 (6.9)	0.011
Normal weight (18.5–23.9)	298 (68.5)	114 (71.2)	0.549	572 (71.3)	222 (64.7)	0.030
Overweight (24.0–27.9)	48 (11.0)	27 (16.9)	0.070	119 (14.8)	82 (23.9)	< 0.001
Obesity (≥ 28.0)	4 (0.9)	5 (3.1)	0.064	14 (1.8)	15 (4.5)	0.013
Multiparity, *n* (%)	133 (30.6)	59 (36.9)	0.166	100 (12.5)	61 (17.7)	0.021
ART use, *n* (%)	71 (16.3)	39 (24.4)	0.032	650 (81.0)	300 (87.6)	0.010
Preexisting hypertension, *n* (%)	6 (1.4)	7 (4.4)	0.050	18 (2.3)	18 (5.3)	0.010
History of GDM, *n* (%)	2 (0.5)	2 (1.3)	0.290	4 (0.5)	2 (0.6)	1.000
PCOS, *n* (%)	5 (1.1)	2 (1.3)	1.000	208 (25.9)	137 (39.9)	< 0.001
Hyperthyroidism, *n* (%)	4 (0.9)	3 (1.9)	0.393	48 (0.6)	2 (0.5)	0.790
Hypothyroidism, *n* (%)	37 (8.5)	10 (6.3)	0.493	76 (9.5)	39 (11.4)	0.135
Gestational weight gain, kg	15.7 (5.9)	17.9 (4.9)	< 0.001	15.5 (5.9)	17.3 (5.1)	< 0.001
Insulin treatment, *n* (%)	—	11 (6.9)	—	—	32 (9.3)	—

Abbreviations: ART, assisted reproductive technology; BMI, body mass index; DC, dichorionic; GDM, gestational diabetes mellitus; MC, monochorionic; PCOS, polycystic ovary syndrome.

### 3.2. Risk Factors for GDM Stratified by Chorionicity

Table 2 presents the risk factors for GDM in twin pregnancies by chorionicity. After incorporating potential risk factors into the multivariable model, maternal age ≥ 35 years (MC: aOR 2.18, 95% CI 1.25–3.81 and DC: aOR 1.32, 95% CI 1.06–1.67) and preexisting hypertension (MC: aOR 2.69, 95% CI 1.04–9.36 and DC: aOR 1.70, 95% CI 1.12–2.59) were risk factors for GDM in both chorionicity groups. Regarding pre‐pregnancy BMI categories, overweight and obesity were significant risk factors for GDM in DC pregnancies (aOR 1.65, 95% CI 1.26–1.98 and aOR 2.31, 95% CI 1.43–3.74, respectively). Multiparity (aOR 1.43, 95% CI 1.10–1.88), ART use (aOR 1.75, 95% CI 1.36–2.26), and complicated with PCOS (aOR 1.98, 95% CI 1.37–4.12) were significant risk factors for GDM in DC pregnancies but not in MC pregnancies.

**Table 2:** Risk factors for GDM in twin pregnancies, stratified by chorionicity.

**Factors**	**MC**		**DC**	
	**cOR (95% CI)**	**aOR (95% CI)** ^**a**^	**cOR (95% CI)**	**aOR (95% CI)** ^**a**^
Maternal age ≥ 35, *n* (%)	2.54 (1.53–4.22)	2.18 (1.25–3.81)	1.52 (1.25–1.56)	1.32 (1.06–1.67)
Maternal BMI				
Normal, reference	—	—	—	—
Underweight, *n* (%)	0.43 (0.24–0.79)	0.46 (0.26–0.92)	0.63 (0.47–0.86)	0.67 (0.50–0.91)
Overweight, *n* (%)	1.47 (0.88–2.47)	1.46 (0.85–2.48)	1.78 (1.45–2.18)	1.65 (1.26–1.98)
Obesity, *n* (%)	3.27 (0.86–12.39)	3.76 (0.98–14.44)	2.74 (1.72–4.37)	2.31 (1.43–3.74)
Multiparity, *n* (%)	1.33 (0.91–1.94)	1.23 (0.81–1.89)	1.32 (1.06–1.65)	1.43 (1.10–1.88)
ART use, *n* (%)	1.65 (1.06–2.57)	1.43 (0.89–2.31)	1.65 (1.31–2.08)	1.75 (1.36–2.26)
Preexisting hypertension, *n* (%)	3.27 (1.08–9.89)	2.69 (1.04–9.36)	2.22 (1.46–3.37)	1.70 (1.12–2.59)
History of GDM, *n* (%)	1.01 (0.98–1.03)	1.02 (0.98–1.05)	1.03 (0.96–1.10)	1.02 (0.96–1.09)
PCOS, *n* (%)	1.04 (0.92–1.15)	1.06 (0.90–1.22)	2.23 (1.64–4.36)	1.98 (1.37–4.12)

Abbreviations: ART, assisted reproductive technology; BMI, body mass index; DC, dichorionic; GDM, gestational diabetes mellitus; MC, monochorionic; PCOS, polycystic ovary syndrome.

^a^Adjustments for maternal age, maternal BMI, multiparity, assisted reproductive technology use, preexisting hypertension, PCOS, and history of GDM.

### 3.3. Maternal and Neonatal Outcomes in MC Twin Pregnancies

Table 3 presents pregnancy outcomes by GDM status in MC pregnancies. For maternal outcomes, GDM was only associated with an increased risk of PE (aOR 1.29, 95% CI 1.04–2.26) after adjusting for confounding factors. There were no statistically significant differences between women with and without GDM regarding GH, ICP, PTD at < 37, < 34, and < 32 weeks, PROM, cesarean delivery, and PPH. For neonatal outcomes, no significant differences were noted in the rate of LGA or SGA neonates. Contrary to theoretical concerns, neonatal hypoglycemia was not increased in the GDM group. Though the proportion of neonates with 5‐min Apgar scores of < 7 and NICU admission rates was higher in the GDM group, these differences did not achieve statistical significance.

**Table 3:** Pregnancy outcomes according to maternal GDM status in monochorionic twin pregnancies.

**Outcomes**	**No GDM**	**GDM**	**cOR (95% CI)**	**aOR (95% CI)**
Mothers	(*n* = 435)	(*n* = 160)		
GH, *n* (%)^a^	8 (1.8)	2 (1.3)	0.68 (0.14–3.22)	0.55 (0.09–3.21)
PE, *n* (%)^b^	43 (9.9)	27 (16.9)	1.29 (1.01–2.37)	1.29 (1.04–2.26)
ICP, *n* (%)^b^	61 (14.0)	23 (14.4)	1.03 (0.61–1.73)	1.13 (0.64–1.99)
PTD < 37 weeks, *n* (%)^c^	294 (67.6)	115 (71.9)	1.23 (0.82–1.83)	1.07 (0.70–1.65)
PTD < 34 weeks, *n* (%)^c^	63 (14.5)	26 (16.3)	1.15 (0.70–1.88)	0.78 (0.43–1.41)
PTD < 32 weeks, *n* (%)^c^	26 (6.0)	5 (3.2)	0.51 (0.19–1.35)	0.36 (0.13–1.33)
PROM, *n* (%)^c^	72 (16.6)	30 (18.8)	1.16 (0.73–1.86)	1.19 (0.73–1.94)
Cesarean delivery, *n* (%)^c^	428 (98.4)	158 (98.8)	1.29 (0.27–6.29)	1.04 (0.19–5.70)
PPH, *n* (%)^c^	11 (2.5)	3 (1.9)	0.73 (0.20–2.67)	0.73 (0.16–3.40)
Neonates	(*n* = 870)	(*n* = 320)		
LGA, *n* (%)^c^	35 (4.0)	11 (3.4)	0.85 (0.40–1.79)	0.71 (0.36–1.40)
SGA, *n* (%)^c^	89 (10.2)	22 (6.9)	0.65 (0.40–1.04)	0.59 (0.34–1.02)
5‐min Apgar score < 7, *n* (%)^c^	4 (0.5)	4 (1.3)	2.74 (0.50–15.09)	2.61 (0.20–34.52)
Neonatal hypoglycemia, *n* (%)^c^	42 (4.8)	12 (3.8)	0.77 (0.36–1.62)	0.66 (0.29–1.47)
NICU admission, *n* (%)^c^	284 (32.6)	121 (37.8)	1.25 (0.88–1.79)	1.15 (0.78–1.71)

Abbreviations: GDM, gestational diabetes mellitus; GH, gestational hypertension; ICP, intrahepatic cholestasis of pregnancy; LGA, large for gestational age; NICU, neonatal intensive care unit; OR, odds ratio; PE, preeclampsia; PPH, postpartum hemorrhage; PROM, premature rupture of membranes; PTD, preterm delivery; SGA, small for gestational age.

^a^Adjustments for maternal age, maternal BMI, multiparity and assisted reproductive technology use, polycystic ovary syndrome, history of gestational diabetes, and insulin treatment.

^b^Adjustments for maternal age, maternal BMI, multiparity, assisted reproductive technology use, polycystic ovary syndrome, history of gestational diabetes, preexisting hypertension, and insulin treatment.

^c^Adjustments for maternal age, maternal BMI, multiparity, assisted reproductive technology use, preexisting hypertension, polycystic ovary syndrome, history of gestational diabetes, hypertensive diseases of pregnancy, insulin treatment, and gestational weight gain.

### 3.4. Maternal and Neonatal Outcomes in DC Twin Pregnancies

Table 4 presents pregnancy outcomes by GDM status in DC pregnancies. For maternal outcomes, GDM was associated with an increased risk of ICP (aOR 1.35, 95% CI 1.09–1.68), PTD at < 37 weeks (aOR 1.13, 95% CI 1.05–1.34) and < 34 weeks (aOR 1.15, 95% CI 1.07–1.75) after adjusting for confounding factors. However, the rate of early PTD at < 32 weeks was comparable between the groups. No differences were found between women with and without GDM in terms of GH, PE, PROM, cesarean delivery, and PPH. For neonatal outcomes, the rates of LGA, SGA neonates, and 5‐min Apgar scores of < 7 were comparable between the GDM and non‐GDM groups. The incidence of neonatal hypoglycemia and NICU admission was not significantly increased in the GDM group.

**Table 4:** Pregnancy outcomes according to maternal GDM status in dichorionic twin pregnancies.

**Outcomes**	**No GDM**	**GDM**	**cOR (95% CI)**	**aOR (95% CI)** ^**a**^
Mothers	(*n* = 802)	(*n* = 343)		
GH, *n* (%)^a^	22 (2.8)	10 (2.8)	0.99 (0.61–1.61)	0.90 (0.52–1.52)
PE, *n* (%)^b^	100 (12.5)	42 (12.3)	0.98 (0.77–1.25)	0.90 (0.67–1.18)
ICP, *n* (%)^b^	121 (15.1)	63 (18.4)	1.27 (1.02–1.56)	1.35 (1.09–1.68)
PTD < 37 weeks, *n* (%)^c^	413 (51.5)	197 (57.3)	1.26 (1.08–1.49)	1.13 (1.05–1.34)
PTD < 34 weeks, *n* (%)^c^	87 (10.9)	49 (14.3)	1.37 (1.08–1.74)	1.15 (1.07–1.75)
PTD < 32 weeks, *n* (%)^c^	33 (4.1)	35 (4.1)	0.99 (0.66–1.48)	0.66 (0.44–1.09)
PROM, *n* (%)^c^	157 (19.6)	14 (19.8)	1.01 (0.83–1.24)	0.96 (0.77–1.18)
Cesarean delivery, *n* (%)^c^	791 (98.6)	338 (98.6)	0.99 (0.50–1.0.96)	0.99 (0.49–2.01)
PPH, *n* (%)^c^	38 (4.7)	19 (5.5)	1.17 (0.82–1.68)	1.11 (0.76–1.63)
Neonates	(n = 1604)	(n = 686)		
LGA, *n* (%)^c^	96 (6.0)	39 (5.7)	0.94 (0.73–1.21)	0.90 (0.71–1.14)
SGA, *n* (%)^c^	98 (6.1)	40 (5.8)	0.95 (0.74–1.23)	1.02 (0.76–1.35)
5‐min Apgar score < 7, *n* (%)^c^	5 (0.3)	2 (0.2)	0.54 (0.15–1.97)	0.46 (0.12–1.75)
Neonatal hypoglycemia, *n* (%)^c^	40 (2.5)	19 (2.8)	1.12 (0.76–1.65)	1.06 (0.70–1.59)
NICU admission, *n* (%)^c^	438 (27.3)	214 (31.2)	1.20 (1.02–1.42)	1.05 (0.88–1.25)

Abbreviations: GDM, gestational diabetes mellitus; GH, gestational hypertension; ICP, intrahepatic cholestasis of pregnancy; LGA, large for gestational age; NICU, neonatal intensive care unit; OR, odds ratio; PE, preeclampsia; PPH, postpartum hemorrhage; PROM, premature rupture of membranes; PTD, preterm delivery; SGA, small for gestational age.

^a^Adjustments for maternal age, maternal BMI, multiparity and assisted reproductive technology use, polycystic ovary syndrome, history of gestational diabetes, and insulin treatment.

^b^Adjustments for maternal age, maternal BMI, multiparity, assisted reproductive technology use, polycystic ovary syndrome, history of gestational diabetes, preexisting hypertension, and insulin treatment.

^c^Adjustments for maternal age, maternal BMI, multiparity, assisted reproductive technology use, preexisting hypertension, polycystic ovary syndrome, history of gestational diabetes, hypertensive diseases of pregnancy, insulin treatment, and gestational weight gain.

## 4. Discussion

Based on a 7‐year retrospective cohort study, we found that the risk factors for GDM varied between MC and DC twin pregnancies. Advanced maternal age and preexisting hypertension were significant risk factors in both types of twin pregnancies, whereas overweight, obesity, multiparity, and ART use were significant risk factors predominantly in DC pregnancies. When comparing the impact of GDM between the two chorionicity groups, we observed that the effects of GDM diverged based on chorionicity. In MC pregnancies, GDM was associated with an increased risk of PE, but it did not significantly link to other maternal or neonatal outcomes. In contrast, DC pregnancies complicated by GDM demonstrated a higher risk of ICP, PTD before 37 and 34 weeks.

The prevalence of GDM in twin pregnancies exhibits substantial heterogeneity across different regions and countries. In western regions, the prevalence ranges from 7.7% to 14.9% [39, 40]. In contrast, Asian and Middle Eastern regions demonstrate markedly higher prevalence rates, with China reporting GDM in 20.4%–29.2% of twin pregnancies [15, 41, 42]. In this study, the overall prevalence of GDM was 28.9%, with a higher prevalence of GDM in DC pregnancies compared with MC pregnancies (30.0% vs 26.9%). This may be attributed to the higher proportion of ART use and advanced maternal age and higher BMI in DC pregnancies, as well as distinct placental characteristics between the two types of twin pregnancies. In DC pregnancies, the presence of two separate placentas may intensify the cumulative effect on insulin resistance, whereas in MC pregnancies, the shared placenta may modulate this effect through different vascular and hormonal adaptations.

Risk factors for GDM in singleton pregnancies are well established, including advanced maternal age, pre‐pregnancy overweight/obesity, family history of diabetes, previous GDM, and PCOS [43–45]. Similar risk factors have also been identified in twin pregnancies [15, 43, 46]. Our findings align with previous studies; however, we further figured that these factors differ by chorionicity. Specifically, overweight, obesity, multiparity, ART use, and complicated with PCOS were identified as specific risk factors for GDM in DC pregnancies, whereas only advanced maternal age and preexisting hypertension were risk factors for GDM in both MC and DC pregnancies. Previous studies have shown that ART procedures and associated hormonal treatments may influence glucose metabolism, thereby increasing the GDM risk [47]. Other risk factors like obesity and PCOS demonstrated not only statistical significance but also substantial effect magnitudes in DC pregnancies, translating to a clinically meaningful more than two‐fold increase in GDM risk. This underscores their importance as primary targets for risk stratification and preventive interventions in this subgroup. In contrast, the higher point estimate for obesity in MC twins suggests a potential, even larger clinical effect, but its wide CI may be attributed to the smaller MC sample size, which needed larger cohorts to clarify it in the future. Future studies with larger cohorts are needed to explore the relationship between obesity and GDM in MC twin pregnancies.

Previous studies examining GDM in twin pregnancies have reported inconsistent findings. Among study populations with higher thresholds for GDM diagnosis, such as Canadian Diabetes Association guideline, GDM was associated with an increased risk of GH, PE, preterm birth, and LGA neonates [20, 21, 23–25, 46], whereas it was linked to a reduced risk of SGA neonates and low Apgar score [21, 23, 24, 40]. Conversely, study populations with lower thresholds for GDM diagnosis, such as the IADPSG criteria, showed no associations between GDM and GH, PE, preterm birth, LGA, and SGA neonates [15, 16]. Only Wen et al. reported a high risk of preterm birth in twins with GDM and reduced risks of SGA neonates in those with only an abnormal fasting glucose level [42].

Our study contributes valuable insights by demonstrating chorionicity‐specific associations, with GDM associated with an increased risk of PE only in MC pregnancies but linked to a higher risk of preterm delivery only in DC pregnancies. These differential impacts may be explained by distinct placental pathophysiology. In MC twins, the shared placenta may amplify the vascular dysfunction associated with GDM, exacerbating endothelial damage and promoting PE through intensified inflammatory cascades [48]. Conversely, the increased placental mass in DC twins creates greater metabolic stress when complicated by higher glucose levels, potentially triggering preterm labor through various mechanisms including heightened inflammation and oxidative stress. The rate of preterm birth was notably high in MC pregnancies, which may mask the potential link between GDM and preterm birth in MC pregnancies.

Our findings have several important implications for clinical practice. First, the high prevalence of GDM in twin pregnancies highlights the need for universal screening and close monitoring of glucose metabolism in this population. Second, the identification of chorionicity‐specific risk factors allows for more targeted surveillance of high‐risk subgroups. For instance, women with DC pregnancies who conceived through ART or have elevated BMI may benefit from earlier or more intensive screening. Third, the chorionicity‐specific outcomes observed suggest that management strategies for GDM in twin pregnancies should be tailored according to chorionicity status. MC twins with GDM may benefit from more intensive monitoring for PE, while DC twins might require closer surveillance for preterm labor. This differentiated approach could optimize maternal and neonatal outcomes in this high‐risk population. Additionally, preparation for potential NICU admission should be considered for DC twin pregnancies affected by GDM.

A major strength of this study is its focus on the under‐researched area of chorionicity‐specific outcomes in twin pregnancies complicated by GDM. The sample size is sufficient to provide robust statistical power for detecting meaningful associations. Additionally, the comprehensive assessment of both maternal and neonatal outcomes allows for a holistic understanding of the impact of GDM in this specific population. However, several limitations should be acknowledged. First, as an observational study, we cannot establish causality between GDM and the observed outcomes, particularly concerning GDM and PE, although Yang et al. reported a causal link between PE and GDM based on a bidirectional Mendelian randomization analysis [49]. Second, despite adjusting for multiple confounders, residual confounding cannot be ruled out. Third, we did not have data on long‐term outcomes for mothers or infants, which limits our understanding of the extended impact of GDM in twin pregnancies. Lastly, the single‐center study design limited the generalizability of our results to other populations. Nonetheless, these results hold clinical value for the specific Western population in China.

## 5. Conclusions

This study demonstrates that the risk factors for GDM and its impact on pregnancy outcomes varied based on chorionicity in twin pregnancies, suggesting that chorionicity should be considered when assessing GDM risk and planning management strategies. Future research should focus on developing chorionicity‐specific guidelines for screening, prevention, and management of GDM in twin pregnancies, as well as investigating the long‐term implications of these findings for maternal and child health.

## Ethics Statement

The study was approved by the Ethics Committee of the First Affiliated Hospital of Chongqing Medical University (No. 201530).

## Consent

Consent was waived due to the retrospective nature of the study and the lack of patient interaction.

## Disclosure

All the authors approved the final manuscript as submitted and agreed to be accountable for all aspects of the work.

## Conflicts of Interest

The authors declare no conflicts of interest.

## Author Contributions

S.H. and H.Q. conceptualized and designed the study; S.H. and X.L. organized the implementation of the study; S.H., X.L., and J.Z. completed all the experimental testing and data collection; S.H., X.L., G.Q., P.L., and L.M. collected the clinical information; S.H. completed the data analysis; S.H. and X.L. drafted the initial manuscript; and H.Q. further refined the manuscript. S.H. and X.L. have contributed equally to this work and co‐first authors.

## Funding

This study was supported by the National Natural Science Foundation of China (10.13039/501100001809, 81520108013) and the Nursing Research Fund of the First Affiliated Hospital of Chongqing Medical University (HLJJ2016‐37).

## Supporting information


**Supporting Information 1** Additional supporting information can be found online in the Supporting Information section. The characteristics of the included participants and excluded participants are presented in Table S1. The characteristics of the study participants stratified by chorionicity are presented in Table S2.

## Data Availability

The datasets used and/or analyzed during the current study are available from the corresponding author upon reasonable request.
