# Supplementary material for: Risk Factors and Pregnancy Outcomes of Twin Pregnancies With Gestational Diabetes Mellitus: A Comparison Based on Chorionicity
Source: J Diabetes Res. 2026 Jan 13;2026:9744892. doi: 10.1155/jdr/9744892 (PMC12800392; doi:10.1155/jdr/9744892)
Supplement: Supplementary file 1 — Supporting Information 1 Additional supporting information can be found online in the Supporting Information section. The characteristics of the included participants and excluded participants are presented in Table S1. The characteristics of the study participants stratified by chorionicity are presented in Table S2. [file JDR-2026-9744892-s001.docx]

Table S1. Characteristics of the included participants and excluded participants

| Variables | Included participants  (n=1740) | Excluded participants  (n=369) | *P* value |
| --- | --- | --- | --- |
| Maternal age, y | 30.9 (3.8) | 30.7 (4.2) | 0.292 |
| ≥35 | 276 (15.9) | 50 (13.6) | 0.269 |
| Pre-pregnancy BMI, kg/m^2^ | 21.6 (2.8) | 21.8 (3.2) | 0.451 |
| Underweight (<18.5), n (%) | 219 (12.6) | 41 (11.1) | 0.115 |
| Normal weight (18.5-23.9), n (%) | 1207 (69.4) | 263 (71.5) |  |
| Overweight (24.0-27.9), n (%) | 275 (15.8) | 49 (13.3) |  |
| Obesity (≥28.0), n (%) | 39 (2.2) | 15 (4.1) |  |
| Multiparity, n (%) | 353 (20.3) | 65 (17.6) | 0.251 |
| ART use, n (%) | 1060 (60.9) | 220 (59.6) | 0.681 |
| Chorionicity | 202 (11.6) |  | 0.082 |
| MC twin pregnancies, n (%) | 595 (34.2) | 144 (39.0) |  |
| DC twin pregnancies, n (%) | 1145 (65.8) | 225 (61.0) |  |
| Preexisting hypertension, n (%) | 50 (2.9) | 14 (3.8) | 0.402 |
| History of GDM, n (%) | 10 (0.6) | 4 (1.1) | 0.286 |
| PCOS, n (%) | 352 (20.2) | 83 (22.5) | 0.357 |
| Hyperthyroidism, n (%) | 14 (0.8) | 5 (0.8) | 1.000 |
| Hypothyroidism, n (%) | 202 (11.6) | 40 (10.8) | 0.720 |
| GDM status, n (%) | 503 (28.9) | 84 (22.8) | **0.018** |
| Insulin treatment, n (%) | 43 (2.5) | 5 (1.4) | 0.249 |

ART, assisted reproductive technology; BMI, body mass index; DC, dichorionic; GDM, gestational diabetes mellitus; MC, monochorionic; PCOS, polycystic ovary syndrome.

Table S2. Characteristics of the study participants stratified by chorionicity

| Variables | MC  (n=595) | DC  (n=1145) | *P* value |
| --- | --- | --- | --- |
| Maternal age, y | 29.5 (4.2) | 31.2 (3.7) | **<0.001** |
| ≥35 | 71 (11.9) | 205 (17.9) | **<0.001** |
| Pre-pregnancy BMI, kg/m^2^ | 21.1 (2.8) | 21.7 (2.8) | **<0.001** |
| Underweight (<18.5), n (%) | 99 (16.6) | 120 (10.5) | **<0.001** |
| Normal weight (18.5-23.9), n (%) | 412 (69.2) | 795 (69.4) | 0.961 |
| Overweight (24.0-27.9), n (%) | 75 (12.6) | 200 (17.5) | **0.003** |
| Obesity (≥28.0), n (%) | 9 (1.5) | 30 (2.6) | 0.141 |
| Multiparity, n (%) | 192 (32.3) | 161 (14.1) | **<0.001** |
| ART use, n (%) | 110 (18.5) | 950 (83.0) | **<0.001** |
| Preexisting hypertension, n (%) | 13 (2.2) | 37 (3.2) | 0.236 |
| History of GDM, n (%) | 4 (0.7) | 6 (0.5) | 0.743 |
| PCOS, n (%) | 7 (1.2) | 345 (30.1) | **<0.001** |
| Hyperthyroidism, n (%) | 7 (1.2) | 7 (0.6) | 0.098 |
| Hypothyroidism, n (%) | 47 (7.9) | 115 (10.1) | 0.110 |
| GDM status, n (%) | 160 (26.9) | 343 (30.0) | 0.166 |
| Fasting glucose, mmol/L | 4.5 (0.5) | 4.5 (0.5) | 0.163 |
| Post-load glucose at 1-h, mmol/L | 8.3 (1.8) | 8.4 (1.8) | **0.049** |
| Post-load glucose at 2-h, mmol/L | 7.0 (1.7) | 7.2 (1.6) | **0.016** |
| Insulin treatment | 11 (1.8) | 32 (2.8) | 0.258 |
| Gestational weight gain, kg | 17.3 (5.2) | 16.8 (5.4) | **0.032** |

ART, assisted reproductive technology; BMI, body mass index; DC, dichorionic; GDM, gestational diabetes mellitus; MC, monochorionic; PCOS, polycystic ovary syndrome.
